# Supplementary material for: TransformEHR: transformer-based encoder-decoder generative model to enhance prediction of disease outcomes using electronic health records
Source: Nat Commun. 2023 Nov 29;14:7857. doi: 10.1038/s41467-023-43715-z (PMC10687211; doi:10.1038/s41467-023-43715-z)
Supplement: Supplementary file 1 — Supplementary Information [file 41467_2023_43715_MOESM1_ESM.pdf]

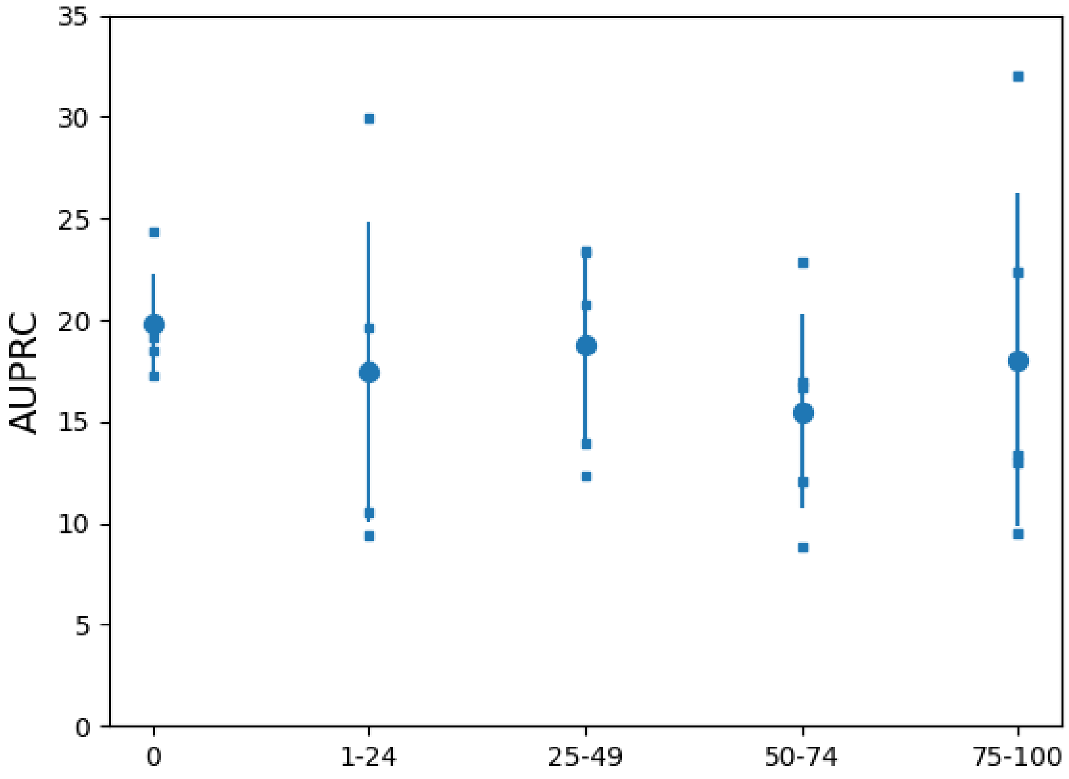

Supplementary Fig.1. Area under the precision-recall curve (AUPRC) of intentional self-harm among PTSD patients (n=70,623) from 1,239 health care facilities. Facilities are grouped by the number of patients in the pretraining cohort. There are 57 patients in a facility on average. The experiment was repeated 5 times with different randomized seeds. Error bars represent standard deviation. The AUPRC is consistent among different hospitals, even for those hospitals which had no patients in the pretraining cohort.

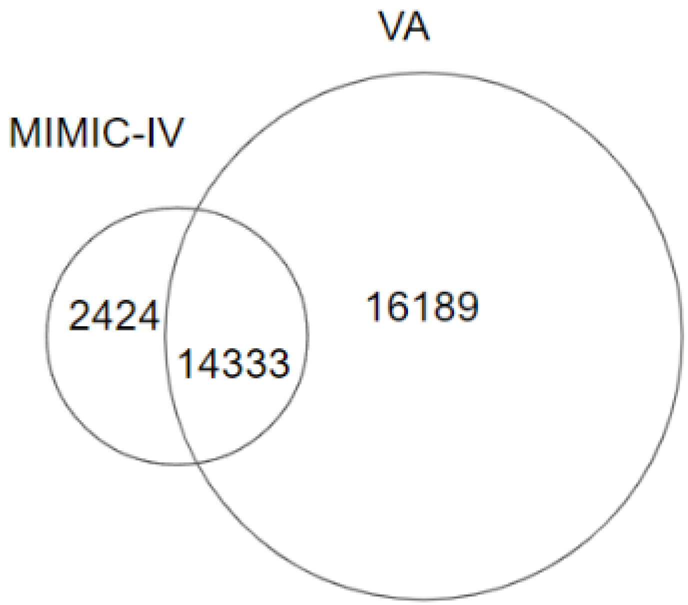

Supplementary Fig.2. Venn diagram on the unique ICD-10-CM codes in the MIMIC-IV data and our VA data. Common codes in MIMIC-IV include: Hypertension(I10), Hyperlipidemia (E785), Gastroesophageal reflux disease (K219), Anxiety (F419), Arteriosclerosis (I2510), Acute kidney failure (N179), Type 2 diabetes mellitus without complications (E119), Hypothyroidism (E039), Obstructive Sleep Apnea (G4733), Unspecified atrial fibrillation (E4891).

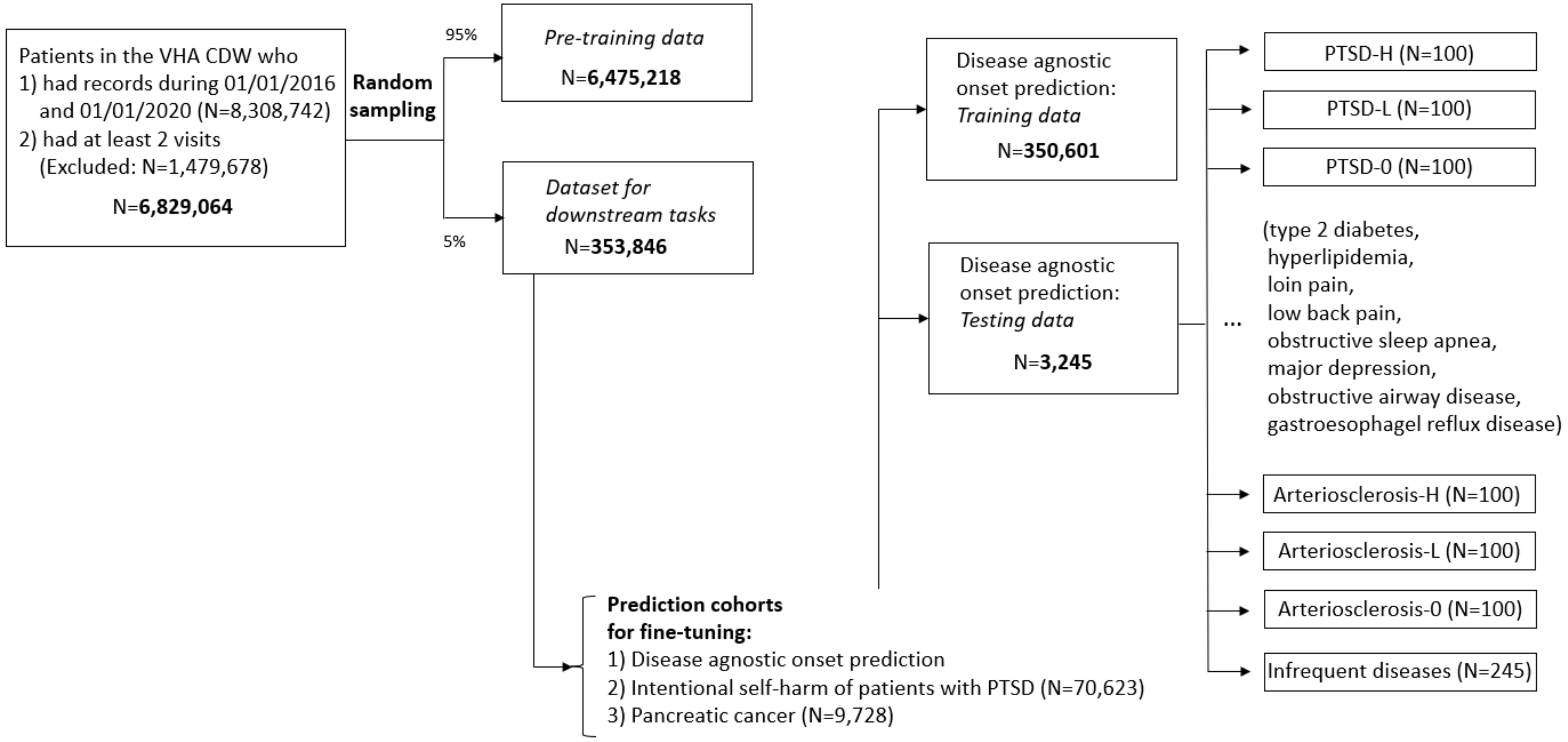

Supplementary Fig.3. Cohort for pretraining and cohort for disease-agnostic onset prediction.

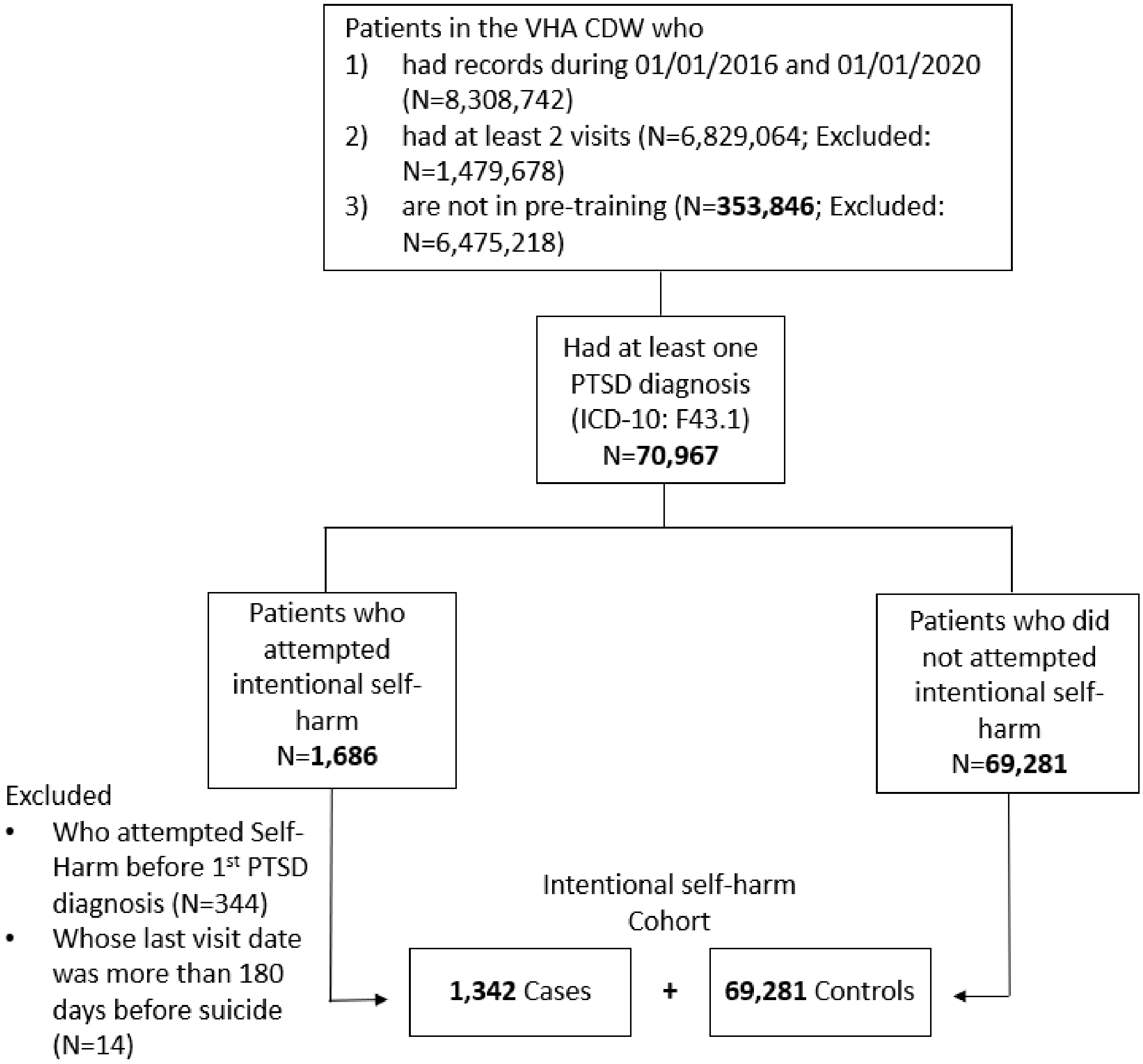

Supplementary Fig.4. Cohort intentional self-harm of patients with PTSD. Intentional self-harms (cases) are selected by patients with codes in Supplementary Dataset 1.

Characteristic	Pretrain		Finetune	
	**Train**	**DOAP**	**Intentional self-harm among PTSD**	**Pancreatic Cancer**
Number of patients	6.5M	353.8k	70.6k	9.7k
Number of visits	255.3M	13.1M	707.0k	105.4k
Number of codes	1.1B	59.2M	3.1M	445.1k

Supplementary Table 1. Statistics on our VHA cohorts. Values are rounded up to the nearest hundreds.

Ablations		/w Code masking		/w Visit masking		/w Visit masking /w Date embedding daysdiff		/w Visit masking /w Date embedding yearmonthdate	
Chronic PTSD	R	81.70	±0.12	83.61	±0.11	83.63	±0.16	83.73	±0.07
	0	77.06	±0.15	78.02	±0.17	78.10	±0.06	77.95	±0.12
Type 2 diabetes	R	85.18	±0.07	85.48	±0.10	85.65	±0.04	85.72	±0.07
	0	80.41	±0.08	81.72	±0.04	81.56	±0.08	81.84	±0.05
Hyperlipidemia	R	87.04	±0.06	87.81	±0.03	88.03	±0.06	88.04	±0.05
	0	81.79	±0.05	83.17	±0.08	83.34	±0.06	83.42	±0.08
Loin pain	R	83.16	±0.10	87.98	±0.04	89.10	±0.06	88.24	±0.05
	0	78.94	±0.12	84.61	±0.12	85.06	±0.06	85.37	±0.08
Low back pain	R	85.78	±0.11	86.70	±0.07	86.93	±0.04	86.94	±0.03
	0	80.68	±0.08	82.21	±0.07	82.27	±0.07	82.30	±0.10
Obstructive sleep apnea	R	81.15	±0.14	82.13	±0.17	82.14	±0.18	82.25	±0.16
	0	73.41	±0.18	74.71	±0.08	74.38	±0.11	74.69	±0.19
Depression	R	87.01	±0.06	87.88	±0.05	87.78	±0.07	87.66	±0.12
	0	82.87	±0.12	83.89	±0.12	83.95	±0.15	83.85	±0.11
Obstructive airway disease	R	84.25	±0.11	86.19	±0.14	86.27	±0.13	86.19	±0.07
	0	77.82	±0.10	80.20	±0.08	80.23	±0.02	80.27	±0.07
Gastroesophageal reflux	R	86.47	±0.20	90.74	±0.28	91.06	±0.25	91.07	±0.11
	0	77.92	±0.28	82.78	±0.36	82.88	±0.14	83.41	±0.33
Arteriosclerosis	R	83.87	±0.14	88.10	±0.06	88.28	±0.13	88.79	±0.10
	0	76.78	±0.13	79.82	±0.08	80.17	±0.09	80.03	±0.20
Uncommon disease/outcome	0	76.64	±0.13	79.66	±0.12	79.98	±0.12	80.11	±0.12

Supplementary Table 2. Ablation study of TransformEHR on disease/outcome agnostic prediction. Our TransformEHR contains three components: 1) encoder-decoder model 2) visit masking 3) date embedding. Mean (standard deviation) of AUROC is reported.

Threshold P	Sensitivity		Specificity		Positive predictive value	
	%	SE	%	SE	%	SE
Logistic regression						
0.1	16.51	±4.14	90.01	±0.00	3.31	±0.83
0.2	33.26	±4.77	80.01	±0.00	3.33	±0.48
0.6	80.23	±2.33	40.01	±0.00	2.67	±0.08
TransformEHR						
0.1	44.88	±2.11	90.01	±0.00	8.80	±0.42
0.2	58.84	±4.47	80.01	±0.00	5.88	±0.45
0.6	94.19	±1.84	40.01	±0.00	3.14	±0.06

Supplementary Table 3. Operating characteristics at a range of thresholds for logistic regression model and our TransformEHR applied in the test data to predict intentional self-harm among PTSD patients.

Characteristic	Group	Unique Patients, No.	Unique Patients, %	AUPRC	
Gender	Male	61,452	87.01%	16.41	±0.42
	Female	9,171	12.99%	15.43	±0.87
Age	18-29	2,425	3.43%	15.01	±0.71
	30-39	13,830	19.58%	16.29	±0.99
	40-49	10,887	15.42%	16.06	±0.26
	50-59	13,126	18.59%	16.03	±0.84
	60-69	18,657	26.42%	16.78	±0.17
	70-79	8,269	11.71%	16.33	±0.27
	80+	3,429	4.86%	18.08	±0.79
Marital Status	Divorced	25,011	35.41%	16.48	±0.55
	Married	18,068	25.58%	16.27	±0.57
	Never Married	21,774	30.83%	16.49	±0.49
	Separated	3,943	5.58%	16.23	±0.65
	Missing	1,827	2.59%	16.04	±0.78
Race	White	47,599	67.39%	16.29	±0.45
	Black	10,765	15.24%	17.17	±0.59
	Hispanic	6,174	8.74%	16.42	±0.40
	Asian	748	1.05%	16.37	±0.42
	Missing	5,337	7.55%	16.12	±0.96

Supplementary Table 4. Socio-demographics and TransformEHR performance with yearmonthdate date embedding: Intentional self-harm cohort. Mean (standard deviation) of AUPRC is reported.

Models	BERT		TransformEHR	
	%	SE	%	SE
Without pretraining	77.37	±1.07	79.36	±0.94
With pretraining	78.31	±0.39	81.15	±0.49

Supplementary Table 5. Generalization evaluation of disease/outcome agnostic prediction: AUROC scores on MIMIC-IV dataset when pretrained from VHA.
