# Supplementary material for: TransformEHR: transformer-based encoder-decoder generative model to enhance prediction of disease outcomes using electronic health records
Source: Nat Commun. 2023 Nov 29;14:7857. doi: 10.1038/s41467-023-43715-z (PMC10687211; doi:10.1038/s41467-023-43715-z)
Supplement: Supplementary file 3 — Description of Additional Supplementary Files [file 41467_2023_43715_MOESM3_ESM.pdf]

File name: Supplementary Data 1

Description: ICD-10 codes used to identify intentional self-harm.
