# Supplementary material for: TransformEHR: transformer-based encoder-decoder generative model to enhance prediction of disease outcomes using electronic health records
Source: Nat Commun. 2023 Nov 29;14:7857. doi: 10.1038/s41467-023-43715-z (PMC10687211; doi:10.1038/s41467-023-43715-z)
Supplement: Supplementary file 5 — Reporting Summary [file 41467_2023_43715_MOESM5_ESM.pdf]

nature portfolio

Corresponding author(s): Hong Yu

Last updated by author(s): Oct 31, 2023

# Reporting Summary

Nature Portfolio wishes to improve the reproducibility of the work that we publish. This form provides structure for consistency and transparency in reporting. For further information on Nature Portfolio policies, see our Editorial Policies and the Editorial Policy Checklist.

## Statistics

For all statistical analyses, confirm that the following items are present in the figure legend, table legend, main text, or Methods section.

| n/a | Confirmed | |
|---|---|---|
| ☐ | ☒ | The exact sample size (*n*) for each experimental group/condition, given as a discrete number and unit of measurement |
| ☐ | ☒ | A statement on whether measurements were taken from distinct samples or whether the same sample was measured repeatedly |
| ☐ | ☒ | The statistical test(s) used AND whether they are one- or two-sided<br>*Only common tests should be described solely by name; describe more complex techniques in the Methods section.* |
| ☒ | ☐ | A description of all covariates tested |
| ☒ | ☐ | A description of any assumptions or corrections, such as tests of normality and adjustment for multiple comparisons |
| ☐ | ☒ | A full description of the statistical parameters including central tendency (e.g. means) or other basic estimates (e.g. regression coefficient) AND variation (e.g. standard deviation) or associated estimates of uncertainty (e.g. confidence intervals) |
| ☐ | ☒ | For null hypothesis testing, the test statistic (e.g. *F*, *t*, *r*) with confidence intervals, effect sizes, degrees of freedom and *P* value noted<br>*Give P values as exact values whenever suitable.* |
| ☒ | ☐ | For Bayesian analysis, information on the choice of priors and Markov chain Monte Carlo settings |
| ☒ | ☐ | For hierarchical and complex designs, identification of the appropriate level for tests and full reporting of outcomes |
| ☒ | ☐ | Estimates of effect sizes (e.g. Cohen's *d*, Pearson's *r*), indicating how they were calculated |

*Our web collection on statistics for biologists contains articles on many of the points above.*

## Software and code

Policy information about availability of computer code

| | |
|---|---|
| Data collection | No software was used for data collection. |
| Data analysis | Our code is publicly available on https://github.com/whaleloops/TransformEHR. Experiments were conducted using Python version 3.8, torch version 1.9.0, transformer library version 4.16.2. Visualization was obtained using Python packages matplotlib version 3.3.2. |

For manuscripts utilizing custom algorithms or software that are central to the research but not yet described in published literature, software must be made available to editors and reviewers. We strongly encourage code deposition in a community repository (e.g. GitHub). See the Nature Portfolio guidelines for submitting code & software for further information.

## Data

Policy information about availability of data

All manuscripts must include a data availability statement. This statement should provide the following information, where applicable:

- Accession codes, unique identifiers, or web links for publicly available datasets
- A description of any restrictions on data availability
- For clinical datasets or third party data, please ensure that the statement adheres to our policy

The study has been approved by the Institutional Review Board at the VA Bedford Healthcare System. The VHA EHR data are available under restricted access for Veterans' privacy and data security laws, access can be obtained by relevant approvals through VA Informatics and Computing Infrastructure (VINCI) (contact: VINCI@va.gov). Individuals who wish to use this data for research purposes must fulfil the research credentialing requirements as outlined by the VA Office of

Research and Development, with the approval process expected to take from 1 month to 1 year.

The MIMIC-IV raw data are publicly available through Physionet. aiming to utilize this data for research will be required to meet research credentialing requirements as outlined at the Physionet's web site: https://physionet.org/content/mimiciv/2.2/.

## Research involving human participants, their data, or biological material

Policy information about studies with human participants or human data. See also policy information about sex, gender (identity/presentation), and sexual orientation and race, ethnicity and racism.

| | |
|---|---|
| Reporting on sex and gender | Statistics are reported in Supplementary Table 4. |
| Reporting on race, ethnicity, or other socially relevant groupings | Statistics are reported in  Supplementary Table 4. |
| Population characteristics | Statistics are reported in Supplementary Table 4. |
| Recruitment | There was no recruitment for this study. The data was collected from the Veterans Health Administrations (VHA) Clinical Data Warehouse (CDW). |
| Ethics oversight | The study protocol was approved by the Institutional Review Board at the VA Bedford Healthcare System. |

Note that full information on the approval of the study protocol must also be provided in the manuscript.

# Field-specific reporting

Please select the one below that is the best fit for your research. If you are not sure, read the appropriate sections before making your selection.

☒ Life sciences ☐ Behavioural & social sciences ☐ Ecological, evolutionary & environmental sciences

For a reference copy of the document with all sections, see nature.com/documents/nr-reporting-summary-flat.pdf

# Life sciences study design

All studies must disclose on these points even when the disclosure is negative.

| | |
|---|---|
| Sample size | Using the Veterans Health Administrations (VHA) Clinical Data Warehouse (CDW), we first identified a total of 8,308,742 patients who received care from more than 1,200 health care facilities of the US VHA from 1/1/2016 to 12/31/2019. For inclusion, we required each patient to have at least two visits: one outcome visit and at least one prior visit to be used for prediction of the outcome. This resulted in a total of 6,829,064 patients. We randomly sampled 353,846 (5%) on encrypted patient identifiers for disease and outcome evaluation.<br>See Supplementary Figure 2 for more detail. |
| Data exclusions | We exclude patient with insufficient number of visits (<2). |
| Replication | Best hyper-parameters in the models were selected from dev data with 50 different searching experiments. Searched hyper-parameters includes: learning rate, normalization weight, and warm up steps. Models were then evaluated on the test data.<br>This process is repeated on each evaluation (DOAP, pancreatic cancer onset prediction, and prediction of Intentional Self-Harm in Patients with PTSD) separately. |
| Randomization | For each disease and outcome prediction, we built training, validation, and test datasets by the ratio of 7:1:2. The training, validation, and test dataset split was performed randomly on encrypted patient identifiers. |
| Blinding | Patient identifiers were encrypted and only used during randomization. |

# Reporting for specific materials, systems and methods

We require information from authors about some types of materials, experimental systems and methods used in many studies. Here, indicate whether each material, system or method listed is relevant to your study. If you are not sure if a list item applies to your research, read the appropriate section before selecting a response.

## Materials & experimental systems

| n/a | Involved in the study |
|---|---|
| ☒ | ☐ Antibodies |
| ☒ | ☐ Eukaryotic cell lines |
| ☒ | ☐ Palaeontology and archaeology |
| ☒ | ☐ Animals and other organisms |
| ☒ | ☐ Clinical data |
| ☒ | ☐ Dual use research of concern |
| ☒ | ☐ Plants |

## Methods

| n/a | Involved in the study |
|---|---|
| ☒ | ☐ ChIP-seq |
| ☒ | ☐ Flow cytometry |
| ☒ | ☐ MRI-based neuroimaging |
